# Extremely Late-Onset Deep Infection Post Inguinal Hernia Repair After Panendoscopy

**DOI:** 10.7759/cureus.22169

**Published:** 2022-02-13

**Authors:** Katrina Ng, Kim Goddard

**Affiliations:** 1 General Surgery, Sir Charles Gairdner Hospital, Perth, AUS; 2 General Surgery, Armadale Health Service, Mount Nasura, AUS

**Keywords:** bacterial translocation, open inguinal hernia repair, delayed infection, abdominal sinus, hernia mesh

## Abstract

Mesh infection after hernia repair is a well-known complication, which can have morbid consequences. This report presents a case of a gentleman with mesh infection many years after initial surgery, potentially from bacterial translocation post-colonoscopy, and describes his successful treatment. This case emphasizes the need to consider mesh infection regardless of time from surgery to presentation.

## Introduction

Inguinal hernia repair is a very common general surgical procedure, with a yearly incidence of 200 per 100,000 in Australia [1], while the lifetime risk of inguinal hernias is 27% in men and 3% in women [2]. The use of mesh is now conventional since 1989 when Lichtenstein published his success of the tension-free hernioplasty [3], but mesh has been used successfully since 1959 when Usher and colleagues demonstrated that Marlex polyethylene made into a monofilament mesh could be used for hernia repairs with low recurrence and infection risk [4]. The risk of surgical site infection after a repair ranges from 3% to 5%, and the risk of deep surgical site infection is even lower at 0.3-0.5% [5]. We present a case of deep infection in a 67-year-old male, 30 years after he underwent bilateral open inguinal hernia repair with mesh. This is the longest interval between mesh placement and infection documented in the literature.

## Case presentation

**Figure 1:**
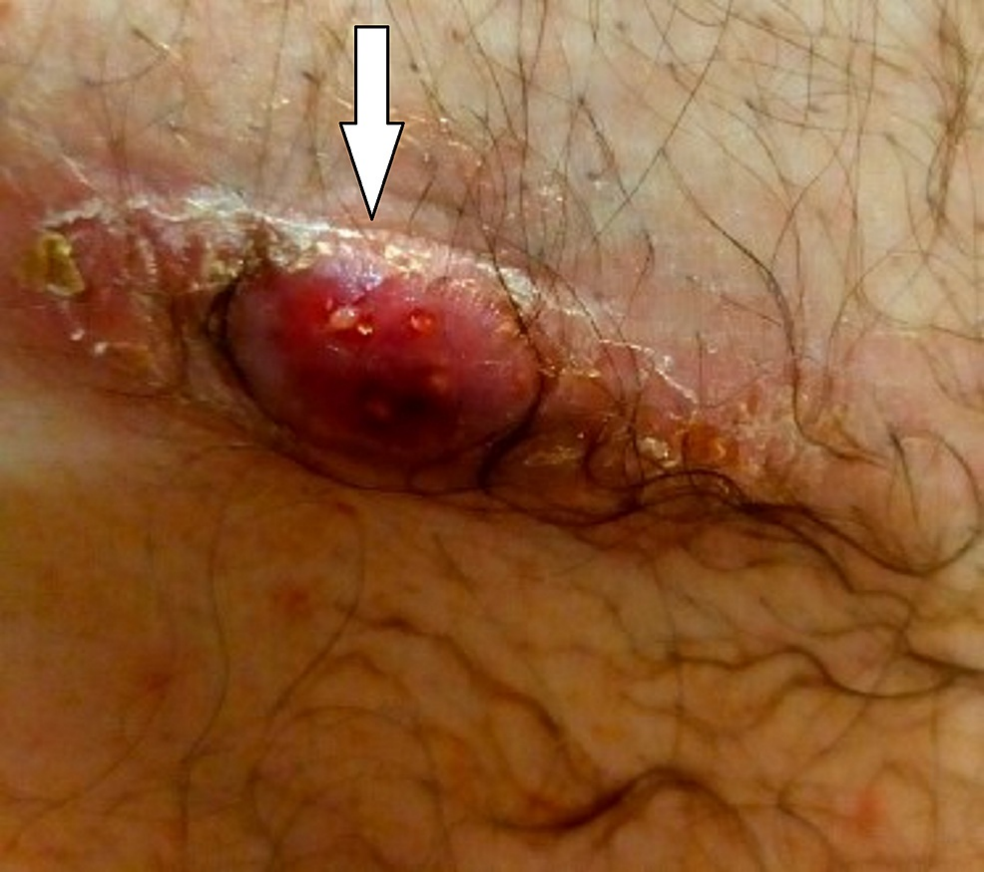
Abscess overlying old surgical scar (arrow pointing to the abscess).

A 67-year-old male patient presented to our hospital’s emergency department with a one-week history of a discharging abscess on the scar of a previous right inguinal hernia repair. He had noted a palpable lump in that region for the past three months, and notably, he had a gastroscopy and colonoscopy for abdominal pain and rectal bleeding two days before the lump appeared. Endoscopy was normal except for mild perianal excoriation. He had no history of trauma. Ultrasound performed five days after he noticed the lump demonstrated a 43 x 23 x 17 mm collection in deep subcutaneous adipose tissue, which was reported as either fat necrosis or a hematoma. As he was pain-free and otherwise well, his general practitioner opted to monitor the lump. His comorbidities included hypertension, dyslipidemia, gastro-oesophageal reflux disease, and mild bronchiectasis. His only previous surgery was bilateral open inguinal hernia repair with mesh 30 years ago. On physical exam, he had a 5 x 1 cm abscess situated on the lateral aspect of the scar from previous right inguinal hernioplasty (Figure 1). There were no groin hernias palpable and the remaining abdominal exam was unremarkable. Swabs taken by his general practitioner grew coliform organisms and methicillin-resistant *Staphylococcus aureus* (MRSA).

He was started on 2 g intravenous ceftriaxone daily, 500 mg intravenous metronidazole twice a day (BD), and 1.25 g intravenous vancomycin BD to cover MRSA. He underwent an MRI of his right groin, which reportedly demonstrated an enterocutaneous fistula, which bifurcated to two loops of the ileum. He then underwent surgical exploration, entry with right Rutherford Morrison incision into the abdominal cavity. There was no bowel adherent to the abdominal wall of the region of interest and no enteric fistula seen. Subcutaneous fat was dissected from the external oblique inferiorly to reach the sinus and further swabs were taken. The sinus was found to track to mesh, and mesh with incorporated muscle layers was excised. The resulting defect was closed in two layers with 0 nylon sutures. Peritoneal and muscle incisions were closed, stapled to the skin. The superficial sinus tract was debrided and left open with a capillary drain in situ. The patient recovered well postoperatively. The wound swab grew methicillin-sensitive *Staphylococcus aureus* (MSSA), and he was stepped down to oral cephalexin for a further 10 days. He was discharged after five days with drain removed prior to discharge, and community wound dressing management was recommended. His open wound was fully healed by secondary intention after three weeks. The patient was followed up seven months later and there was no recurrence of the hernia.

## Discussion

Tension-free inguinal hernia repair is the gold standard as it has the lowest recurrence rate and is the predominant type of repair. A rare but potential complication, however, is deep infection with mesh involvement and is sometimes seen long after the repair, sometimes years later [6]. The longest time between repair and infection reported in the literature is 17 years [6], and case series range from four months to 10 years [6,7]. Range of causes has been attributed to this complication, such as remnant contamination from primary surgery and formation of biofilm [8], bacterial translocation secondary to septic events, and de-novo infection from fistula formation, whether with intestine or through a transcutaneous tract [7]. In this case, considering the patient never had an issue for 30 years, it is unlikely that the mesh was infected from the primary repair. He had a colonoscopy two days prior to abscess formation, and while it cannot be proven that this was the etiology, the risk of bacteremia following colonoscopy is around 4.4% [9]. This could be from bacteremia following intravenous cannulation, or probably less likely from bacterial translocation from the gut. The growth of coliform bacteria from his initial swab supports this hypothesis. The other agent, *Staphylococcus aureus*, is a common skin and gut commensal organism. There have been isolated case reports of orthopedic prosthesis infection post endoscopy, and 25 cases of infective endocarditis in the literature [10]. In the meantime, consequential bacteremia from intravenous cannulation is much more common, with 30-40,000 catheter-related bloodstream infections in the US and an incidence of 0.2% for peripheral lines [11].

A combination of medical and surgical approaches, such as antimicrobial therapy and removal of mesh, has been shown to be effective in treating deep infections [6,7,12,13], and conservative management with antibiotics usually fails. In cases where only antibiotics and/or radiologically guided drainage of the collection was performed, this failed and the patient required surgical debridement and removal of mesh eventually [14,15]. However, there was a case series where mesh could be salvaged after surgical wound debridement and continuous irrigation through a drain if polyester or polypropylene mesh was used. Expanded polytetrafluoroethylene (ePTFE) patches could not be salvaged and had to be removed, and this was thought to be due to its microporous construction [16]. It is not known what mesh was used in this case. Failure of conservative management could be secondary to the fibroblastic reaction of the bacteria with the mesh, forming a thick capsule, which is a barrier for antimicrobials to penetrate the mesh. *Staphylococcal aureus*, the most common organism implicated in mesh infection, is known to produce a biofilm protecting it from the host’s immunological response and from antibiotics [14].

## Conclusions

We report the latest known onset of delayed deep mesh infection, which was successfully treated with a combination of antimicrobial agents and surgical debridement and removal of the mesh. The patient reports that the wound is completely healed with no signs of infection or hernia recurrence seven months after mesh removal.
